# Absence of Posterior Vitreous Detachment Is a Risk Factor of Severe Bleb-Related Endophthalmitis

**DOI:** 10.1155/2019/1585830

**Published:** 2019-01-14

**Authors:** Kazuhiko Umazume, Jun Suzuki, Yoshihiko Usui, Katsuhiko Maruyama, Yoshihiro Wakabayashi, Hiroshi Goto

**Affiliations:** Department of Ophthalmology, Tokyo Medical University, Tokyo, Japan

## Abstract

**Purpose:**

Bleb-related endophthalmitis (BRE) after glaucoma surgery is an infection caused by bacteria in the avascular bleb gaining access into the eye. We report the clinical features and outcome of 10 consecutive eyes with severe BRE treated at our hospital.

**Methods:**

Ten patients (10 eyes) with stage IIIb BRE after trabeculectomy diagnosed and treated at the Department of Ophthalmology, Tokyo Medical University, between April 2013 and May 2015, were studied. Patient background, type of glaucoma, interval from the first trabeculectomy, pretreatment bleb findings, causative microorganisms, surgical methods, status of posterior vitreous detachment (PVD), and postoperative visual acuity were examined.

**Results:**

The 10 patients comprised 8 males and 2 females, with mean age of 70.6 years at BRE onset. The types of glaucoma were open-angle glaucoma in 7 patients, neovascular glaucoma in 2, and secondary glaucoma in 1. All eyes underwent trabeculectomy combined with mitomycin C prior to the development of BRE. The interval from the first glaucoma surgery to onset of endophthalmitis was 8.5 ± 4.1 years. Examination of the bleb revealed leakage of aqueous humor from the avascular bleb in all eyes. Bacteria were isolated from intraocular samples of 8 eyes; namely, *Viridans streptococci* in 5 eyes, *Staphylococcus epidermidis* in 1, *Branhamella catarrhalis* in 1, and coagulase-negative Staphylococci in 1. BRE was treated by vitrectomy in 9 eyes and enucleation in 1 eye. PVD was produced intentionally during vitrectomy in 6 eyes. Histopathological examination of the enucleated eye showed no PVD. Visual acuity improved by 3 lines or more in 6 patients, while decimal visual acuity remained lower than 0.1 in 4 patients.

**Conclusion:**

BRE developed frequently in eyes with no PVD. The absence of PVD may be a risk factor of severe BRE.

## 1. Introduction

Bleb-related endophthalmitis (BRE) that develops after glaucoma surgery is an infection caused by bacteria in the avascular bleb gaining access into the eye. BRE is a disease with poor visual outcome. Visual acuity is maintained at 20/400 or better in 22 to 57% of eyes after onset of endophthalmitis [1]. BRE is classified into several stages: stage I is blebitis, stage II is endophthalmitis involving mainly the anterior chamber, and stages IIIa and IIIb are endophthalmitis involving the vitreous [2]. A multicenter 5-year follow-up study in Japan reported an overall incidence of bleb-related endophthalmitis (all stages) of 2.2%, with 48% of the cases in stage I, 29% in stage II, 9% in stage IIIa, and 14% in stage IIIb. [3] The incidence of BRE in other countries has been reported to range from 0.45 to 1.1% [4–6] and is lower compared to that in Japan. A reason for the higher incidence in Japan is due to the use of trabeculectomy with mitomycin C in all the cases surveyed. Although the use of mitomycin C, an antimetabolite, improves bleb survival and maintains a low filtering bleb [7], the resulting bleb tends to have a thin wall and few blood vessels. As a result, the risk of bleb leakage and infection is increased. There is a concern that the incidence of late-onset BRE will increase in the future [8]. For the treatment of BRE, while frequent instillation of antibiotic eye drops and intravitreal antibiotic injection can be used up to stage IIIa, vitrectomy is required when the endophthalmitis reaches stage IIIb [9]. According to previous reports, development of stage IIIb BRE is very rare, with an incidence of 0.08 to 0.3% [3, 4].

In infectious endophthalmitis including BRE, it is known that invasion and proliferation of bacteria and fungi inside the eye exacerbates inflammation [10, 11]. Previously, we studied endogenous endophthalmitis caused by translocation of bacteria or fungi from other organs into the intraocular space. When we performed vitrectomy on patients with endogenous endophthalmitis, we found that posterior vitreous detachment (PVD) was absent in 80% of the eyes [12]. When formed vitreous is in contact with the posterior pole, the vitreous body acts as a culture medium for invading microorganisms, which probably increases the severity of endophthalmitis.

In the present study, we studied 10 consecutive cases of severe BRE (stage IIIb) which is a rare disease. We report the clinical characteristics, outcome, and status of posterior vitreous detachment in these eyes.

## 2. Methods

Ten consecutive patients (10 eyes) diagnosed with severe BRE (stage IIIb) at the Tokyo Medical University Hospital between April 2013 and May 2015 were studied. The patients comprised 8 males and 2 females with the mean age of 70.6 ± 12.4 years at onset of BRE. The glaucoma types were primary open-angle glaucoma (POAG) in 7 patients, neovascular glaucoma secondary to central retinal vein occlusion in 2 patients, and secondary glaucoma associated with uveitis in 1 patient (Table 1).

The items examined were causative microorganisms, history of surgery before onset of endophthalmitis, clinical findings, chronic use of antibiotic eye drops, surgical method, status of posterior vitreous detachment (PVD), and pre- and postoperative visual acuity. Clinical findings focused on avascular bleb, leakage of aqueous humor from the filtering bleb, fibrin deposition, hypopyon, and intraocular lens (IOL) implantation. Posterior vitreous detachment was defined as detachment of the vitreous body around the optic disc or in the vascular arcade.

All vitrectomies were conducted using a 25-gauge Constellation Vision System (Alcon Laboratories Inc.). Balanced salt solution (BSS) containing ceftazidime (20 mg/ml) and vancomycin (1 mg/ml) was used as the irrigation solution. For isolation and identification of causative microorganisms, the vitreous fluid sample was collected during vitrectomy and used in culture, smear, and microscopic examinations.

### 2.1. Statistical Analyses

Data are expressed as mean ± standard deviation. Preoperative and postoperative visual acuity was compared using the Student's *t*-test. Statistical analysis was performed using MedCalc statistical software version 12.1.1 (MedCalc software, Mariakerke, Belgium). A *p* value less than 0.05 was considered significant.

## 3. Results

Bacteria were isolated from vitreous samples collected during vitrectomy or from the enucleated eye in 8 of 10 eyes. The bacteria were identified as *Viridans streptococci* in 5 eyes, *Branhamella catarrhalis* in 1 eye, *Staphylococcus epidermidis* in 1 eye, and coagulase-negative Staphylococci in 1 eye. No bacteria were detected in 2 eyes (Table 2).

The surgical history (multiple surgeries in some eyes before onset of endophthalmitis) was trabeculectomy + phacoemulsification and aspiration + IOL implantation in 5 eyes, trabeculectomy alone in 5 eyes, trabeculectomy following phacoemulsification and aspiration + vitrectomy in 1 eye. One patient performed trabeculectomy twice. All trabeculectomies were fornix-based with mitomycin C augmentation. The mean interval from the last trabeculectomy to onset of endophthalmitis was 8.5 ± 4.1 (range 4–15) years. At presentation to the Department of Ophthalmology, the mean visual acuity (logMAR) was 2.46 ± 0.74, and mean intraocular pressure was 14.0 ± 10.5 mmHg. Examination of the anterior chamber revealed avascular bleb, leakage of aqueous humor from the filtering bleb, and fibrin deposition in all eyes, hypopyon in 4 eyes, and IOL implantation in 6 eyes. In this study, chronic use of antibiotics, defined as continuous use for 1 year prior to onset of BRE, was found in 6 patients (60%).

In one patient who presented with no light perception and severe pain, enucleation of the eyeball was conducted (Table 1). In the remaining 9 eyes, BRE was treated by IOL removal + vitrectomy in 3 eyes, vitrectomy alone in 3 eyes, and phacoemulsification without IOL plantation and aspiration + vitrectomy in 3 eyes, including repeat vitrectomy in 1 eye to resolve infection.

**Figure 1:**
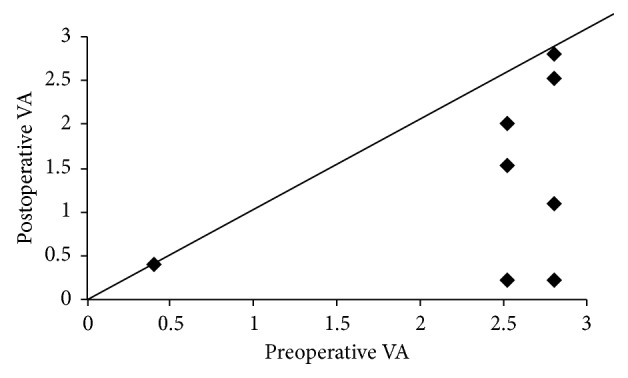
Comparison of pre- and postoperative visual acuity in 9 eyes treated for bleb-related endophthalmitis.

The changes in visual acuity from before to after treatment for BRE in 9 eyes are shown in Figure 1. When visual improvement was defined as a decrease in logMAR of 0.3 or more, posttreatment visual acuity was improved in 6 eyes and unchanged in 3 eyes, with no deterioration. This result confirmed that vitrectomy for BRE was effective to a certain extent in improving or maintaining visual acuity.

**Figure 2:**
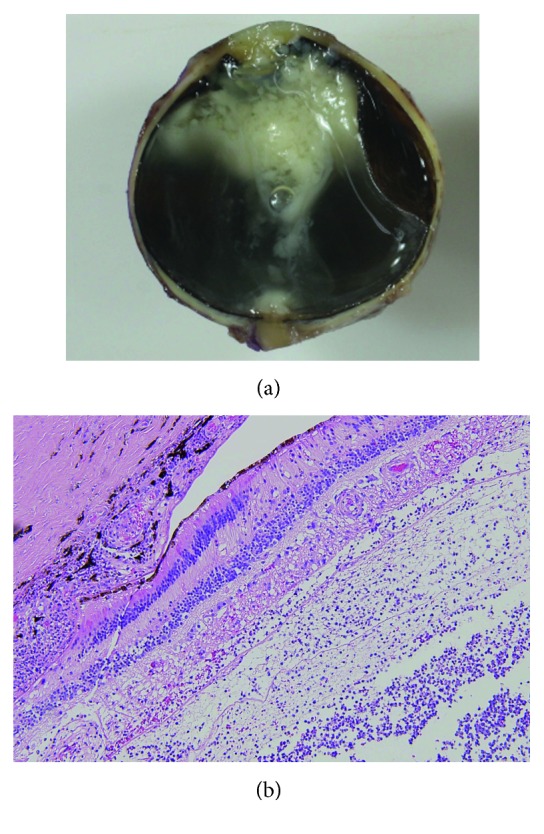
Absence of posterior vitreous detachment in the enucleated eye. (a) Sagittal section and (b) hematoxylin-eosin staining.

Among 9 eyes that underwent vitrectomy, PVD was produced during vitrectomy in 6 eyes. The mean age of patients in whom PVD had occurred before vitrectomy (73.0 ± 16.6 years) was not different from the age of those without PVD (69.2 ± 12.7 years) (Table 3). In 1 eye that was enucleated because of severe pain and no light perception at presentation, examination of the enucleated eye also showed no PVD (Figure 2).

## 4. Discussion

Bleb-related endophthalmitis occurring with late onset after glaucoma surgery is the postoperative complication that results in the worst outcome. According to the Collaborative Bleb-related Infection Incidence and Treatment Study (CBIITS) conducted in Japan, the incidence of bleb-related infection that progressed to stage IIIb endophthalmitis in a 5-year follow-up was 0.31% [3]. In the present study, 6 of 9 eyes with BRE treated with vitrectomy achieved improvement of visual acuity of three lines or more. While this finding shows a certain degree of effectiveness of vitrectomy for BRE, it also confirms that BRE remains a disease with poor visual outcome. Various risk factors for the development of BRE have been reported, including nasal and inferior blebs [13], leakage from the filtering bleb and hypotony [8, 14], limbus-based trabeculectomy [15], aphakic eye, IOL implantation [1, 3], and chronic use of eye drops [16].

In endophthalmitis that occurs after cataract surgery, because the most frequent causative microorganism is *Staphylococcus epidermidis*, a less virulent bacteria species, the visual outcome after endophthalmitis is relatively favorable [17, 18]. For early-onset BRE that develops within 6 weeks after glaucoma surgery, *Staphylococcus epidermidis* is also known to be a frequent causative bacterial species, as in endophthalmitis after cataract surgery. On the other hand, BRE that develops later than 6 weeks after glaucoma surgery is often caused by more virulent microorganisms such as *Streptococcus* species and Gram-negative bacteria, with markedly poorer visual outcome [1, 19]. In the present study also, *Viridans streptococci* were isolated from 50% of the cases. However, less virulent bacteria or normal microflora such as coagulase-negative Staphylococci and *Staphylococcus epidermidis* also caused severe endophthalmitis in our study.

In a previous study, we found that PVD was absent in 8 of 10 consecutive eyes in which vitrectomy was performed for endogenous endophthalmitis [12]. In the present series of BRE, we also found that PVD did not occur spontaneously in 7 of 10 eyes. Moreover, in one enucleated eye, the absence of PVD was confirmed by histopathological examination. The mean age of BRE onset in all 10 patients (70.6 ± 12.4 years) was not significantly different from that of the PVD-negative patients (69.2 ± 8.4 years). Our patients were older than the reported mean age of spontaneous occurrence of PVD (54 to 57 years). Therefore, we speculate that the presence or absence of PVD may be related to the pathogenesis of endophthalmitis. A previous study indicated a relation between the presence of vitreous body and proliferation of bacteria [20]. In addition, the severity of endophthalmitis is known to depend on the virulence of the causative bacteria. Our findings that even bacteria with low virulence and normal microflora caused severe BRE and that PVD was absent even at an old age suggest that the vitreous body may be involved not only in proliferation but also in migration for causative microorganisms. It is possible that in the presence of the vitreous body, bacteria that have gained access into the eye from the aqueous humor leakage site of the avascular bleb can be transmitted through the vitreous body to the retina, facilitating the establishment of retinal infection. Upon reaching the posterior segment of the eye, transmission may occur through the Cloquet's canal [21] or via the posterior vitreous cortex in contact with the retina, although these mechanisms remain to be elucidated. In other words, the absence of PVD may be a risk factor of severe BRE. In the future, with an increase in number of patients who undergo trabeculectomy with mitomycin C and aging of these patients, there is a concern that the incidence of BRE may increase. Currently, visualization of the vitreous body by optical coherence tomography has become possible. Regular follow-up for the presence or absence of PVD may be necessary in patients who have undergone trabeculectomy. In addition, the possibility of using vitreolytic agents such as ocriplasmin [22] to prevent onset of BRE has to be examined.

The present study confirms the absence of PVD in a large proportion of eyes with severe BRE and suggests a relationship between the absence of PVD and pathogenesis of endophthalmitis.

## Figures and Tables

**Table 1:** Clinical data of patients with bleb-related endophthalmitis.

Case	Age (years)	Gender	Type of glaucoma	Treatment	Preoperative VA	Postoperative VA	Postlectomy (years)	Avascular bleb	Bleb leakage	Chronic use of antibiotics
1	75	Male	SG (uveitis)	Vit	HM	HM	8	+	+	+
2	80	Male	NVG	IOL removal + Vit	HM	20/2000	6	+	+	−
3	82	Male	NVG	PEA-Vit	20/50	20/50	9	+	+	+
4	53	Female	POAG	Vit	HM	20/30	10	+	+	+
5	72	Male	POAG	Enucleation	sl(−)	sl(−)	15	+	+	+
6	83	Male	POAG	Vit	HM	HM	16	+	+	+
7	64	Male	POAG	IOL removal + Vit	HM	0.6	4	+	+	−
8	54	Male	POAG	PEA-Vit	HM	20/30	6	+	+	+
9	85	Female	POAG	IOL removal + Vit	HM	20/30	5	+	+	−
10	58	Male	POAG	PEA + Vit	sl(+)	20/200	6	+	+	−

SG, secondary glaucoma; NVG, neovascular glaucoma; POAG, primary open-angle glaucoma; Vit; vitrectomy; PEA, phacoemulsification and aspiration; IOL, intraocuar lens; VA, visual acuity; HM, hand motion; sl, sensus luminis.

**Table 2:** Culture results of vitreous samples.

Causative bacteria	Number of eyes
Bacteria detected	8
*Viridans streptococci*	5
*Branhamella catarrhalis*	1
*Staphylococcus epidermidis*	1
Coagulase-negative Staphylococci	1
No bacteria detected	2

**Table 3:** Ages of patients with and without posterior vitreous detachment at vitrectomy.

	Number	Age (mean ± standard deviation)
PVD(−)	6	69.2 ± 12.7
PVD(+)	3	73.0 ± 16.6

PVD; posterior vitreous detachment.

## Data Availability

All the data supporting the results are shown in the paper and can be available by request from the corresponding author.

## References

[1] Song A., Scott I. U., Flynn M. P. H. H. W., Budenz D. L. (2002). Delayed-onset bleb-associated endophthalmitis. *Ophthalmology*.

[2] Yamamoto T., Kuwayama Y. (2011). Interim clinical outcomes in the collaborative bleb-related infection incidence and treatment study. *Ophthalmology*.

[3] Yamamoto T., Sawada A., Mayama C. (2014). The 5-year incidence of bleb-related infection and its risk factors after filtering surgeries with adjunctive mitomycin C. *Ophthalmology*.

[4] Wallin Ö., Al-ahramy A. M., Lundström M., Montan P. (2013). Endophthalmitis and severe blebitis following trabeculectomy. Epidemiology and risk factors; a single-centre retrospective study. *Acta Ophthalmologica*.

[5] Zahid S., Musch D. C., Niziol L. M., Lichter P. R., Collaborative G. (2013). Risk of endophthalmitis and other long-term complications of trabeculectomy in the collaborative initial glaucoma treatment study (CIGTS). *American Journal of Ophthalmology*.

[6] Vaziri K., Kishor K., Schwartz S. G. (2015). Incidence of bleb-associated endophthalmitis in the United States. *Clinical Ophthalmology*.

[7] Yamamoto T., Kuwayama Y., Nomura E. (2013). Changes in visual acuity and intra-ocular pressure following bleb-related infection: the Japan Glaucoma Society Survey of bleb-related infection report 2. *Acta Ophthalmologica*.

[8] Jampel H. D., Quigley H. A., Kerrigan-Baumrind L. A. (2001). Risk factors for late-onset infection following glaucoma filtration surgery. *Archives of Ophthalmology*.

[9] Yassin S. A. (2015). Bleb-related infection revisited: a literature review. *Acta Ophthalmologica*.

[10] Gentile R. C., Shukla S., Shah M. (2014). Microbiological spectrum and antibiotic sensitivity in endophthalmitis. *Ophthalmology*.

[11] Schimel A. M., Miller D., Flynn H. W. (2013). Endophthalmitis isolates and antibiotic susceptibilities: a 10-year review of culture-proven cases. *American Journal of Ophthalmology*.

[12] Umazume K., Suzuki J., Usui Y., Wakabayashi Y., Goto H. (2016). Possible relation between lack of posterior vitreous detachment and severe endogenous endophthalmitis. *Journal of Ophthalmology*.

[13] Caronia R. M., Liebmann J. M., Friedman R., Cohen H., Ritch R. (1996). Trabeculectomy at the inferior limbus. *Archives of Ophthalmology*.

[14] Solus J. F., Jampel H. D., Tracey P. A. (2012). Comparison of limbus-based and fornix-based trabeculectomy: success, bleb-related complications, and bleb morphology. *Ophthalmology*.

[15] Rai P., Kotecha A., Kaltsos K. (2012). Changing trends in the incidence of bleb-related infection in trabeculectomy. *British Journal of Ophthalmology*.

[16] Mochizuki K., Jikihara S., Ando Y., Hori N., Yamamoto T., Kitazawa Y. (1997). Incidence of delayed onset infection after trabeculectomy with adjunctive mitomycin C or 5-fluorouracil treatment. *British Journal of Ophthalmology*.

[17] Katz L. J., Cantor L. B., Spaeth G. L. (1985). Complications of surgery in glaucoma. *Ophthalmology*.

[18] Lalwani G. A., Flynn H. W., Scott I. U. (2008). Acute-onset endophthalmitis after clear corneal cataract surgery (1996-2005). Clinical features, causative organisms, and visual acuity outcomes. *Ophthalmology*.

[19] Moloney T. P., Park J. (2014). Microbiological isolates and antibiotic sensitivities in culture-proven endophthalmitis: a 15-year review. *British Journal of Ophthalmology*.

[20] Meredith T. A., Aguilar H. E., Miller M. J., Gardner S. K., Trabelsi A., Wilson L. A. (1990). Comparative treatment of experimental Staphylococcus epidermidis endophthalmitis. *Archives of Ophthalmology*.

[21] Kishi S. (2016). Vitreous anatomy and the vitreomacular correlation. *Japanese Journal of Ophthalmology*.

[22] Haller J. A., Stalmans P., Benz M. S. (2015). Efficacy of intravitreal ocriplasmin for treatment of vitreomacular adhesion. *Ophthalmology*.

